# The Effect of Silicon Phase Morphology on Microstructure and Properties of AlSi10Mg Alloys Fabricated by Selective Laser Melting

**DOI:** 10.3390/ma15248786

**Published:** 2022-12-09

**Authors:** Liyun Wu, Zhanyong Zhao, Peikang Bai, Zhen Zhang, Yuxin Li, Minjie Liang, Wenbo Du

**Affiliations:** 1 Department of Mechanical Engineering, Taiyuan Institute of Technology, Taiyuan 030008, China; 2 School of Materials Science and Engineering, North University of China, Taiyuan 030051, China; 3 College of Materials Science and Engineering, Taiyuan University of Science and Technology, Taiyuan 030051, China; 4 National Key Laboratory for Remanufacturing, Academy of Army Armored Forces, Beijing 100072, China

**Keywords:** selective laser melting, AlSi10Mg, microstructure, mechanical properties, corrosion performance

## Abstract

This paper investigated the effect of silicon phase morphology and size on microstructure, mechanical properties, and corrosion resistance of the AlSi10Mg alloys fabricated by selective laser melting (SLM). Using different heat treatment conditions for SLM-fabricated alloys, the microstructure characteristics and mechanical properties are analyzed. The corrosion behavior analysis is also performed using potentiodynamic polarization, electrochemical and immersion tests. Results show that the AlSi10Mg alloy directly fabricated by SLM has a continuous eutectic silicon network, which has a small driving force for corrosion and facilitates the deposition of corrosion products and generates a dense protective film. On the contrary, the formation of large isolated and uniformly distributed silicon particles produces a greater corrosion driving force after heat treatment, which makes most of the corrosion products transfer to the solution. The corrosion resistance of AlSi10Mg alloy directly fabricated by SLM is better than that of the alloys with heat treatment. Moreover, the heat treatment reduces the hardness of AlSi10Mg alloys due to the decrease in the solid solution strengthening effect.

## 1. Introduction

The development of selective laser melting (SLM) technology gradually replaces the traditional casting process to prepare complexed Al-Si alloy components. Compared with the casting process, the SLM process overcomes the brittle, coarse and needle-like eutectic Si structure, which improves the mechanical performance [1,2]. In the preparation of aluminum alloys using SLM technology, the most popular composition is the near-eutectic AlSi10Mg alloy. As SLM technology has the characteristics of fast heating rate and fast cooling rate (10^6^~10^8^ K/s), the microstructure is composed of ultra-fine metastable cell structures, and the fabricated components have excellent mechanical properties [3,4,5]. However, the high cooling rate during the SLM forming process results in the composition difference and internal stress. Therefore, post-processing is necessary for the SLM fabricated components to adjust the composition and eliminate stress. Several studies have reported the development of a heat treatment process for optimizing the SLM-fabricated AlSi10Mg alloy based on that used for casting but have not reached a general consensus yet. Annealing [6,7], solution treatment [7,8] and solution + aging treatment [6,8] can decrease the tensile strength and fatigue strength, and also increase the ductility of AlSi10Mg alloy. Fiocchi [9] claimed that long-time low-temperature annealing could reduce the hardness of the alloy. Considering the differences in solidification mode, thermal gradient and microstructure of AlSi10Mg alloy prepared by SLM or casting, the conventional heat treatment might be not suitable or the best treatment method for SLM. Although conventional heat treatment can enhance the mechanical and corrosion properties of casted AlSi10Mg alloy, it has a negative effect on the mechanical properties of SLM AlSi10Mg alloy [10,11,12].

The marine and shipbuilding industry is one of the fastest-growing industries for SLM technology applications. Based on the advantages of Al-Si alloy, it has received extensive attention. At the same time, the research shows that the corrosion resistance of Al-Si alloy prepared by SLM technology is better than that of conventional technology [13]. Although the SLM-prepared AlSi10Mg is a well-studied material, most reported works focus on studying the effect of optimizing the SLM technological parameter and the effect of heat treatment on mechanical properties. Few works investigate the heat treatment design and corrosion performance of SLM-fabricated AlSi10Mg alloys. Alghamdi et al. [14] reported that the influence of different cooling rates, that is, water quenching (WQ), air cooling (AC) and furnace cooling (FC), on the microstructure and mechanical properties of the SLM fabricated AlSi10Mg kept at 520 ℃ for 1 h. Results showed the directly SLM-prepared alloy had the highest hardness, while FC heat treatment reduced the hardness of alloys due to the occurrence of spheroidization and coarsening of silicon particles. Chen et al. [15] investigated the corrosion resistance of different planes of SLM-prepared Al-12Si alloy and found that the XY plane (a plane perpendicular to the building direction) had a better corrosion resistance than the XZ plane (a plane parallel to the building direction). The main reason is that the Si network on the XY plane benefits the growth of corrosion products. Yang et al. [13] compared the corrosion resistance between casted Al-12Si alloy and SLM fabricated Al-12Si alloy, and found the latter had a good corrosion resistance due to its smaller size of the eutectic silicon particles. Leon et al. [16] investigated the surface roughness effect of the SLM-fabricated AlSi10Mg components on the corrosion behavior and corrosion fatigue strength. Results illustrated that the corrosion resistance and corrosion fatigue life of the polished SLM samples were improved. In other words, the poor corrosion resistance is due to the increase in surface roughness caused by a large number of inherent cavities and other surface defects in the SLM fabrication. Revilla et al. [17] used SEM and SKPFM techniques to explore the local corrosion behavior of AlSi10Mg samples manufactured by additive manufacturing technology. According to the morphological characteristics of the corrosion area, a larger and rougher microstructure appeared at the molten pool boundary than that in other areas. The corrosion often occurs at the molten pool boundary since the driving force is larger. However, more investigations should be made to adjust and control the heat treatment for SLM-fabricated AlSi10Mg alloy, and the corresponding corrosion mechanism is still unclear.

In this paper, the microstructure evolution and the corresponding corrosion mechanism of SLM-prepared AlSi10Mg alloys are investigated via different heat treatments at various times. The corrosion behaviors of AlSi10Mg alloy directly fabricated by SLM and alloys with different heat treatments are studied through the electrochemical test, the impedance spectroscopy test (EIS) and immersion tests. According to the electrochemical principles, the relationships between the corrosion properties of AlSi10Mg alloy and the size and morphology of the silicon phase are analyzed, and the underlying corrosion mechanism of AlSi10Mg alloy in NaCl solution is revealed. It assists in the establishment of a general corrosion mechanism and provides some new values for the applications of SLM-fabricated AlSil0Mg alloys in marine environments.

## 2. Materials and Methods

**Figure 1:**
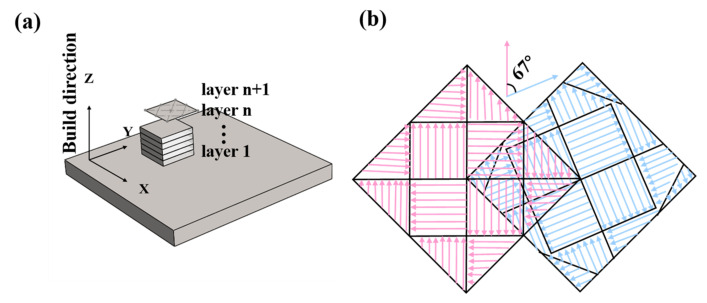
(**a**) Schematic diagram of processing, (**b**) schematic diagram of scanning strategy.

The alloys were fabricated by a Renishaw AM250 system using a selective laser melting process. Table 1 shows the chemical composite of the AlSi10Mg powder (wt.%) used in the experiment. Table 2. shows the parameters of the SLM process. The preheating temperature of the printed substrate is 200 °C. The laser scanning rotates 67° between the scanning strategy of successive layers to obtain a better overlap. The schematic diagram of the processing process and the scanning strategy are shown in Figure 1a,b. Before the SLM forming experiment, the powder was dried in an XMTD-8222 vacuum drying oven for 2 h at the temperature of 100 °C. Argon was used as a protective gas to prevent the preparation process from oxidizing. The size of the fabricated alloy is 10 mm × 10 mm × 10 mm. The SLM-fabricated alloy was heated to 500 °C at a rate of 10 °C/min for different holding times, followed by quenching in water [18,19]. The detailed holding heat treatment times are given in Table 3, where the alloys are named alloys A, B, C, D and E, respectively.

Metallographic morphology analysis was firstly conducted by the optical microscope, then the microstructure and composition of alloys were characterized by scanning electron microscope (SEM) (Zeiss Ultra 55, Jena, Germany) equipped with energy spectrum analysis (EDS). The corrosive liquid used in the test is Keller’s etchant, which ratio is 2.5vol%HNO_3_ + 1vol%HF + 95vol%H_2_O + 1.5vol%HCl. The corrosion time was 15 s. Electron backscattered diffraction (EBSD) was performed on a field emission scanning electron microscope (JEOL JSM-7800F) equipped with the Channel-5 analysis software package. The step size was 2 μm on all the EBSD scans.

The hardness of different samples was measured by the JMHVS-1000AT precision automatic turret digital display at a load of 0.1 kg and an indentation time of 15 s. The hardness values of each sample come from five different points on the same plane.

The alloys in electrochemical tests were prepared to the size of 10 mm × 10 mm × 3 mm. The alloys were first polished with 400#–2000# silicon carbide sandpaper, ultrasonically cleaned and dried. Then, they were connected to the copper wire and sealed with epoxy resin, only leaving an effective working area of 1 cm^2^. When the epoxy resin is cured, the alloys were polished, ultrasonically cleaned and dried again for testing. Electrochemical tests were conducted using an electrochemical workstation (CHI660E) at room temperature. The Tafel polarization curve test and Electrochemical impedance spectroscopy test were performed on the Alloy A and Alloys B~E. The conventional three-electrode battery system, consisting of a platinum electrode, a saturated calomel electrode (SCE) as reference electrode and a working electrode (test alloy), was used as a test system. The test solution used in this experiment is a 3.5 wt.% NaCl solution prepared with 18.2 MΩ·cm deionized water. The scanning rate of the potentiodynamic polarization curve was 0.1 mV/s within a range of the obtained open-circuit potential (OCP) value ±400 mV/SCE. The electrochemical impedance spectroscopy (EIS) measurements were performed with a scanning frequency range of 100 kHz to 10 mHz and a voltage disturbance amplitude of 10 mV. Before polarization and EIS measurements, all the samples were immersed in NaCl solution to obtain a relatively stable open circuit potential (OCP). The ZSimpWin software was used to fit and analyze the EIS data. The immersion test was conducted by suspending each alloy in a prepared 3.5% NaCl solution. According to a certain test period, each alloy was taken out to observe the surface morphology and cross-section morphology.

## 3. Results

### 3.1. Microstructural Analysis

**Figure 2:**
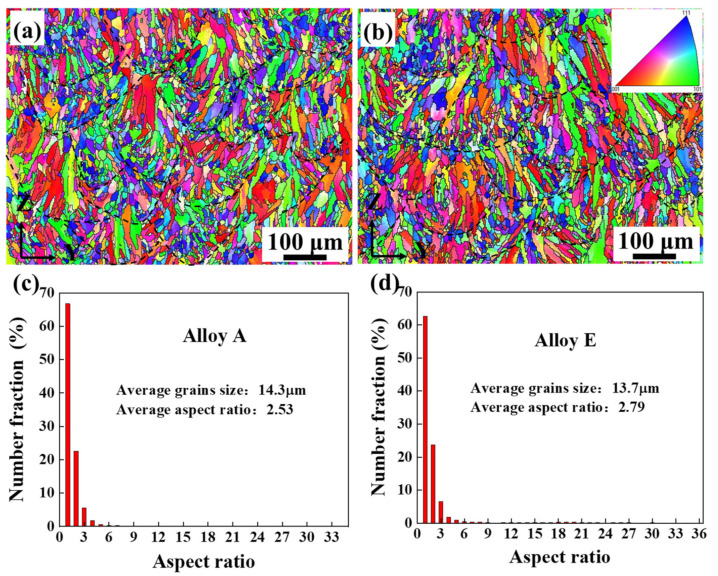
EBSD inverse pole figure (IPF)maps and the corresponding aspect ratio distribution: (**a**,**c**) Alloy A, (**b**,**d**) Alloy E.

Figure 2 shows the inverse pole figures (IPF) and the aspect ratio distribution of Alloy A and Alloy E in parallel to the build direction. The overlapping fan-shaped molten pools, as marked by black dashed lines, are caused by the remelting between channels and layers. As shown in Figure 2a, the molten pools are mainly composed of columnar grains and equiaxed grains. Its interior is dominated by columnar grains, while the boundary is dominated by fine equiaxed crystals. A similar morphology is observed in Figure 2b. The definition of grain aspect ratio is $\varphi =L_{2}/L_{1}$, where the long axis and short axis of grains are$\;L_{2}\;$and $L_{1}$, respectively. When φ> 3.3, the grain is considered to be columnar grains [17]. As shown in Figure 2c, there exist a large proportion of columnar crystals, and the columnar grains of Alloy A account for 8.3%. The average width of the columnar grains is 1.37 µm, and the average size of the equiaxed grains is 13.1 µm. The proportion of columnar grains in Alloy E is 10.8%, where the average width of the columnar grains is 0.89 µm, and the average size of the equiaxed grains is 12.6 µm, as shown in Figure 2d. Comparing Alloy A with E, the heat treatment at 500 °C × 5 h does not change the grain size distinctively.

**Figure 3:**
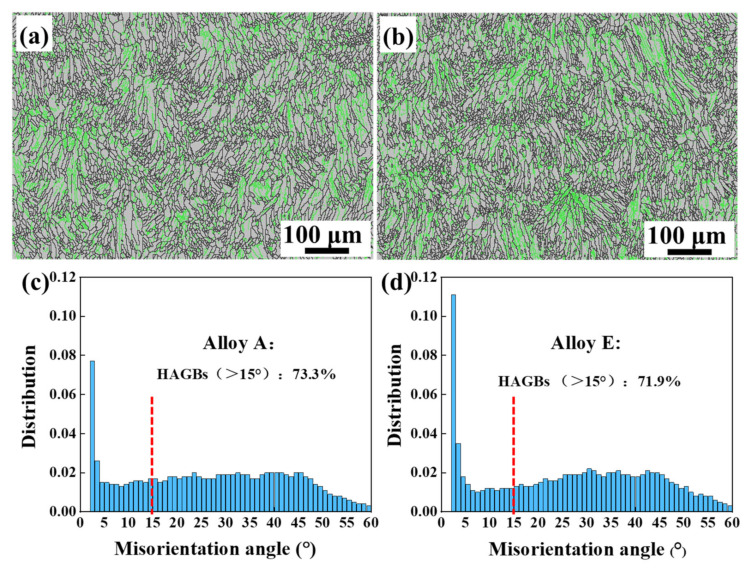
EBSD grain boundary misorientation maps and statistical analysis: (**a**,**c**) Alloy A, (**b**,**d**) Alloy E.

Figure 3 illustrates the distribution and the statistical of grain boundary misorientation of Alloys A and E. Generally, angles of grain boundary misorientation larger than 15° are defined as high-angle grain boundaries (HAGB), while they are defined as low-angle grain boundaries (LAGB) if they are smaller than 15°. As shown in Figure 3, the HAGB (marked in black) is distributed in the center of the molten pool, and the LAGB (marked in green) is distributed on the boundary of the molten pool. When the manufacturing of a new track (or layer) was completed, the previously solidified track was partially or completely remelted again due to the irradiation of the high-energy laser beam. This phenomenon is similar to tempering heat treatment, which leads to recrystallization and then the formation of HAGB. However, due to the rapid movement of the laser beam during SLM processing, the rapid melting and solidification of the matrix occur. The sub-grains located at grain boundaries first appear in the SLM-fabricated AlSi10Mg components [20]. The grain boundaries are first in the pattern of LAGB and then transform into HAGB because of the heat input [20,21,22]. However, the rapid remelting and solidification cannot achieve complete recrystallization, which produces some LAGB at the grain boundary [23]. According to the EBSD statistical analysis, the proportions of the LAGB of Alloys A and E are 73.3% and 71.9%, respectively. Thus, there is no distinctive difference in the distribution of HAGB of AlSi10Mg alloys fabricated by SLM before and after heat treatment at 500 °C.

**Figure 4:**
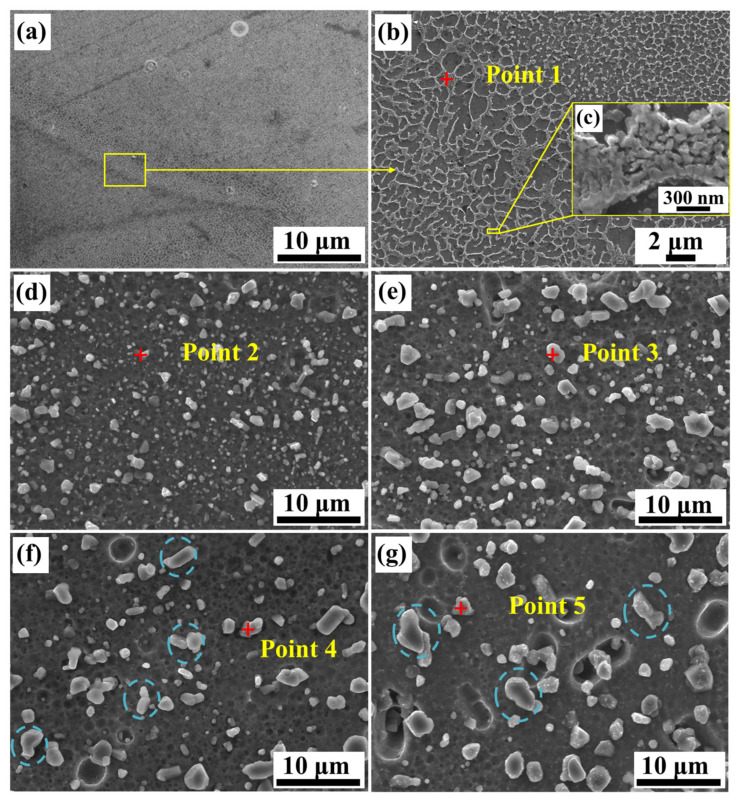
The SEM micrographs of the SLM fabricated AlSi10Mg alloy under different conditions: (**a**) the overlap profile of the molten pool of Alloy A, (**b**) the magnification of Alloy A in (**a**), (**c**) further magnification of networked eutectic silicon of Alloy A, (**d**) Alloy B (**e**) Alloy C, (**f**) Alloy D, (**g**) Alloy E.

Figure 4a shows the microstructure of Alloy A along the building direction, where the overlapping profile of the molten pool is clearly observed. Figure 4b is an enlargement of Figure 4a, which illustrates the overlap area and non-lap area with significant differences. The microstructure is composed of a dark gray cell phase and a light gray network structure. According to the EDS result of point 1 in Table 4, the light gray network structure is eutectic Si, and the dark gray cellular phase is α-Al. The network eutectic Si embedded in the boundaries of α-Al cellular is composed of fine spherical silicon particles with a diameter of about ~200 nm, as shown in Figure 4c. Figure 4d–g show the microstructure evolution of Alloy B, C, D and E. The main secondary phase is the Si phase in the alloys (Points 2, 3, 4, 5) from the EDS analysis, as listed in Table 4. As the time of heat treatment increases, the outline of the molten pool and the continuous eutectic silicon network gradually disappear, while the homogeneously and independently distributed spherical Si particles are produced. As time further increases, the homogeneously distributed microstructure gradually becomes coarser, where the size of silicon particles increases from a few hundred of nanometers (Figure 4c) to a few microns (Figure 4g). In Figure 4f,g, the overlapping phenomenon of silicon particles appears, then they are merging and grow up (marked by a blue dotted circle).

### 3.2. Microhardness

**Figure 5:**
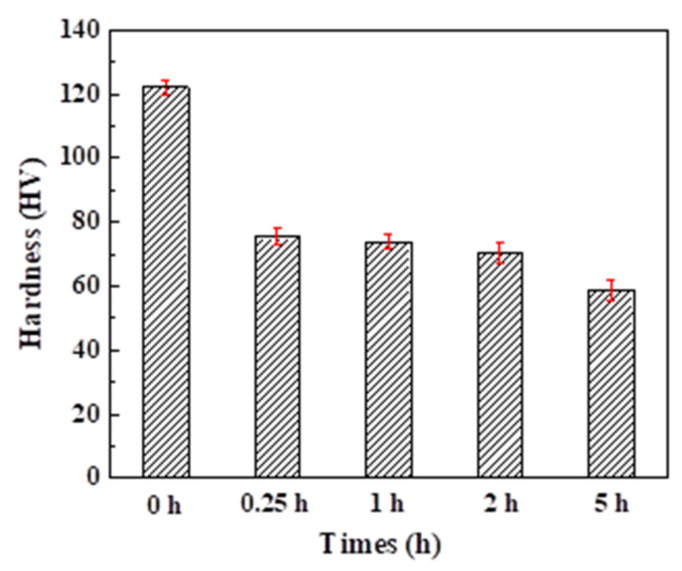
The Vickers hardness of the AlSi10Mg sample fabricated by SLM and the heat-treated samples.

The microhardness distributions of alloys A–E are shown in Figure 5, where Alloy A has the highest microhardness of 122.25 HV. The microhardness of Alloy B significantly decreases down to only 75.36 HV with a decrease rate of 38.4%. As the heat-treatment time further increases, the microhardness of alloys shows a decreasing trend, where Alloy E only has half the microhardness of Alloy A.

### 3.3. Corrosion Behavior Analysis

#### 3.3.1. Potentiodynamic Polarization Tests

**Figure 6:**
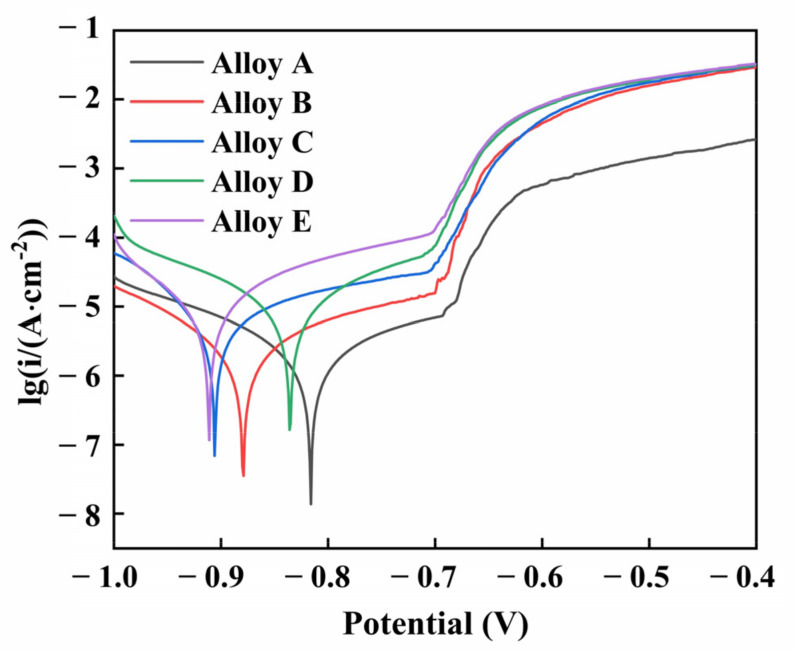
Potentiodynamic polarization curves of Alloys A, B, C, D and E in the aerated 3.5 wt.% NaCl solution.

Figure 6 presents the potentiodynamic polarization curves of Alloys A, B, C, D and E in the aerated 3.5 wt.% NaCl solution. The silicon phase morphology has a great effect on the anodic polarization curve, while the influence on the cathodic polarization curve is relatively small. The increase in the passive current density, as the increase of heat treatment time. Production of pitting breakdown potential shows a slight negative shift. Table 5 lists the fitting parameters for each alloy. The corrosion rate of Alloy A is the lowest (3.39 × 10^−7^ A·cm^−2^), and its corresponding corrosion resistance is the best due to a stable passivation film. As the silicon particles grow, the corrosion current density increases from 6.92 × 10^−7^ A·cm^−2^ to 5.01 × 10^−6^ A·cm^−2^, which means that the protective effect of the passivation film gradually weakens. Therefore, the growth of silicon particles accelerates the corrosion of AlSi10Mg alloys fabricated by SLM after heat treatment, which deteriorates the corrosion resistance of AlSi10Mg alloys with heat treatment.

**Figure 7:**
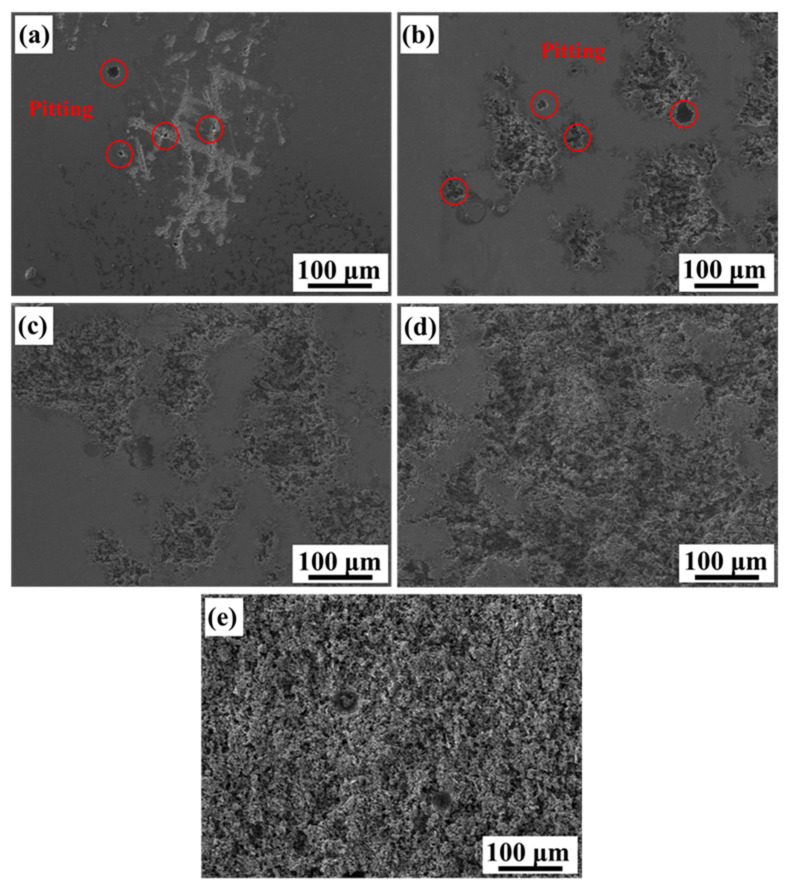
SEM images of the alloys after polarization experiments in the aerated 3.5 wt.% NaCl solution: (**a**) Alloy A, (**b**) Alloy B, (**c**) Alloy C, (**d**) Alloy D, (**e**) Alloy E.

Figure 7 displays the surface corrosion morphology of each alloy after the potentiodynamic polarization test. Alloy A has small pitting corrosion and local slight corrosion at the boundary of the molten pool, as shown in Figure 7a. The pitting holes of Alloy B distinctively became larger and start diffusing around, as shown in Figure 7b. With the silicon particles coarsening, the pitting sensitivity increases, and it is gradually transformed into local corrosion, as shown in Figure 7c,d. For the alloy with the heat treatment of 5 h (Alloy E), its surface is completely destroyed, resulting in extensive corrosion, as shown in Figure 7e.

#### 3.3.2. EIS Measurement

**Figure 8:**
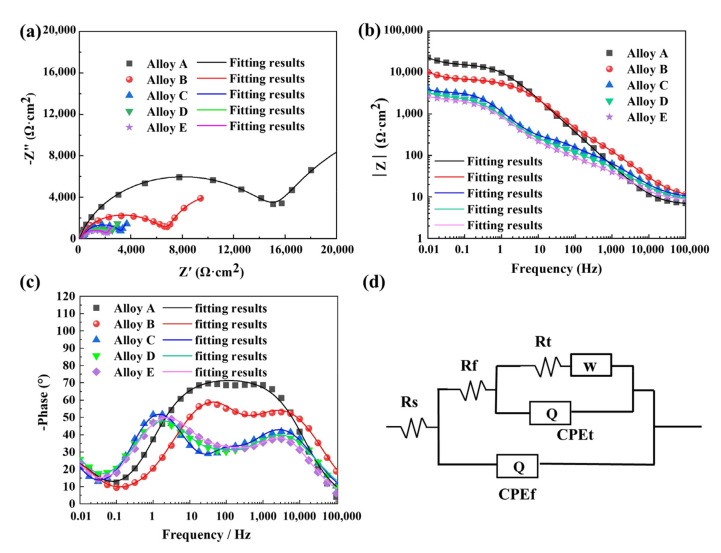
Electrochemical impedance spectra of the alloys A-E in 3.5 wt.% NaCl solution: (**a**) Nyquist, (**b**) Bode, (**c**) Bode, (**d**) Equivalent circuit.

Figure 8 compares the electrochemical impedance spectroscopy (EIS) of alloys A–E in 3.5 wt.% NaCl solution under open circuit potential and the equivalent circuit. The fitting quality is evaluated by the chi-square value (χ2), which is the square of the standard deviation between the original data of EIS and the measured data of EIS [24]. The chi-square values are less than 1 × 10^−3^ for all fitting results of the equivalent circuit. Figure 8a presents the Nyquist plots of alloys A, B, C, D and E, where their changing trends are similar to each other. They are composed of a high-frequency capacitive arc and a low-frequency Warburg line, indicating that the corrosion mechanism is controlled by the charge transfer process and the diffusion process. Since a larger diameter of the semi-circular arc means a better corrosion resistance of the alloy [25], the diameter of the semi-circular arc from Alloy A to Alloy E decreases, which means that their corrosion resistance gradually becomes weaker with increasing the time of heat treatment. This is consistent with the result of the potentiodynamic polarization tests. With the growth of silicon particles, the capacitance arc radius of each alloy gradually becomes smaller, which indicates a descending order of corrosion resistance: A > B > C > D > E. Figure 8b,c show the Bode plots of alloys A, B, C, D, and E. In Figure 8b, the impedance modulus in the high-frequency region (F > 1 kHz) represents the electrolyte resistance [26,27], and the impedance modulus in the low-frequency region (F < 1 Hz) represents the total resistance of the existing electrode system, which can quickly evaluate the corrosion resistance of the alloy [26,28]. The low-frequency modulus of Alloy A is the largest, while the modulus of the alloy decreases in turn as the heat treatment time increases. As indicated in Figure 8c, two time constants exist in the Bode plots (phase angle). Therefore, the equivalent circuit, including a corrosion product layer and an electric double layer, is used to analyze the corrosion process and reflects the dynamic characteristics of the alloy electrode system, as shown in Figure 8d. The equivalent circuit (EC) consists of solution resistance ($R_{s}$), the capacitance component ($Q_{f}$), the film resistance ($R_{f}$) and charge transfer resistance ($R_{t}$). The other capacitance component ($Q_{t}$) is connected in parallel with $R_{t}$, and the diffusion element ω is connected in series with $R_{t}$. In order to obtain detailed information about the non-ideal dielectric properties of each alloy, a constant phase element (CPE) is used instead of a pure capacitive element (Q) to characterize surface unevenness, surface roughness, impurities, etc. The CPE impedance is defined by [29]:

(1) $$Z_{CPE}={\left[Y{\left(jw\right)}^{n}\right]}^{-1}$$

where, *Y* is the admittance with the unit is $\Omega^{-1}cm^{-2}S^{n}$, and j is the imaginary number. ω=2πf is the angular frequency and f is the frequency, and *n* is the empirical index of CPE with a range of −1≤n≤1. These variables are related to surface roughness and uneven current distribution caused by surface defects [30]. Furthermore, there is an inverse relationship between polarization resistance and corrosion current density. Polarization resistance ($R_{p}$) is often used to investigate the change in corrosion rate, which is a combination of alloy electrode charge transfer resistance ($R_{t}$) and oxide film resistance ($R_{f}$). The polarization resistance of this equivalent circuit is:$\;R_{p}=R_{t}+R_{f}$. Table 6 lists the EIS fitting results of each alloy, where the $R_{p}$ of Alloy A is largest at 60.82 kΩ∙cm^2^. With the growth of silicon particles, the $R_{P}$ are 16.73 kΩ∙cm^2^, 5.03 kΩ∙cm^2^, 4.04 kΩ∙cm^2^, and 3.18 kΩ∙cm^2^, respectively. Because of the reduction of film resistance and charge transfer resistance, the alloys after heat treatment are more susceptible to Cl^−^ corrosion in NaCl solution, which has a negative effect on the corrosion behavior.

#### 3.3.3. Immersion Test

**Figure 9:**
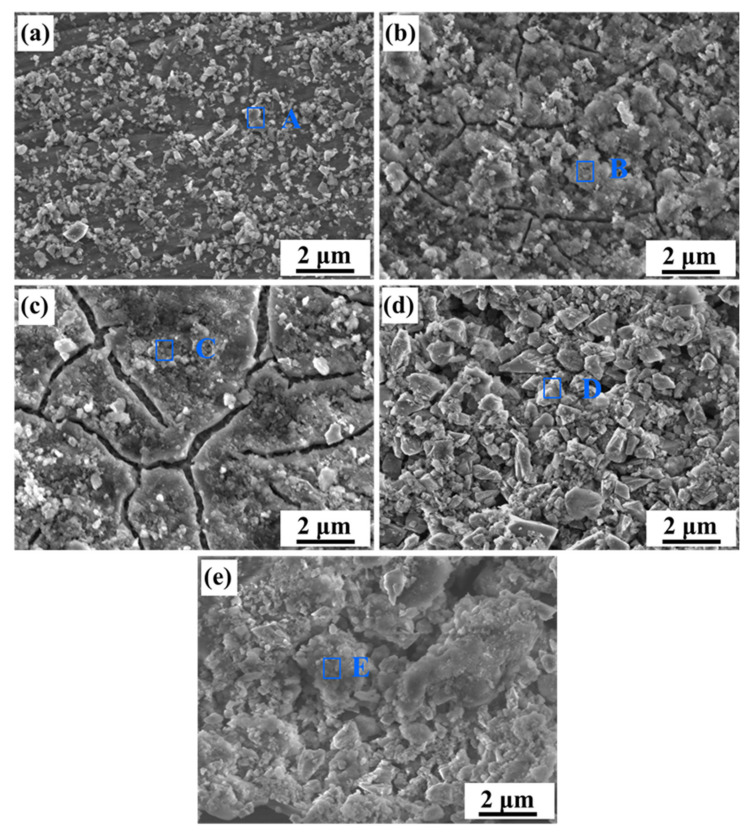
Corrosion morphologies of the alloys A–E after immersion in the aerate 3.5 wt.%NaCl solution: (**a**) Alloy A, (**b**) Alloy B, (**c**) Alloy C, (**d**) Alloy D, (**e**) Alloy E.

Figure 9 compares the corrosion morphologies of alloys A–E after the immersion test. What stands out in this figure are the obvious corrosion products. As shown in Figure 9a, the corrosion products in the pattern of thin and light debris are uniformly deposited on the surface of Alloy A. While the corrosion products of Alloys B and C are loose blocks and propagated along the microcrack, which divides the layer into some 2–6 µm fragments, as shown in Figure 9b,c. The cracks on these corrosion products are caused by dehydration during the drying process. Figure 9d,e are the corrosion product morphologies of Alloys D and E, which gradually become loose and porous. For the corrosion products at the interface Al-Si alloy, the below reactions mainly occur [31]:

Anodic reaction: Al → Al^3+^ (aq) + 3e^−^ (2)

Cathodic reaction: 2H_2_O + 2e^−^ → H_2_(g) + 2OH^−^(aq) (3)

Products formation: Al^3+^ +3OH^−^ → Al(OH)_3_ (4)

Overall reaction: 2Al + 6H_2_O → 2Al (OH)_3_ + 3H_2_ (5)

The Al(OH)_3_ on the alloys is quickly dehydrated and an Al_2_O_3_ protective film is generated, which provides an effective protection [32]. Table 7 lists the results of the EDS point scanning analysis, which demonstrate that the corrosion products are rich in oxygen elements, indicating that Al_2_O_3_ are the main corrosion products on the alloy surface.

**Figure 10:**
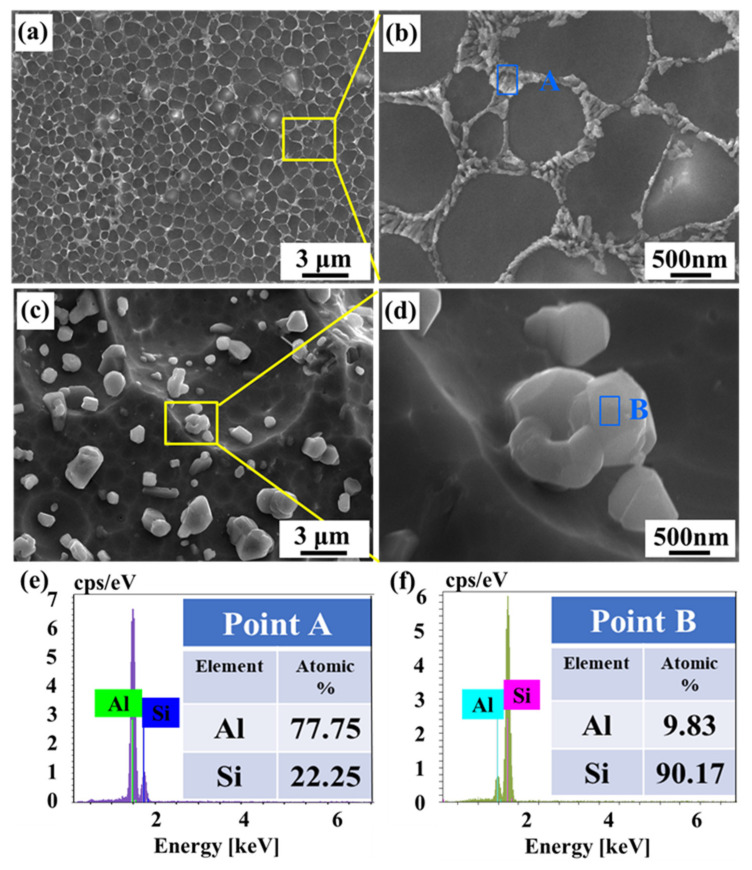
SEM images of surface morphologies after removing corrosion production: (**a**,**b**) Alloy A, (**c**,**d**) Alloy E, (**e**,**f**) EDS point scan analysis results of points A and B.

In order to further understand the influence of the size and distribution of the silicon phase on the corrosion behavior of the alloy, Figure 10 displays the morphology after removing the corrosion products. As shown in Figure 10a, the morphology of Alloy A has a continuous eutectic silicon network, and its surrounding Al matrix is corroded away. Figure 10b is a partial enlarged view of Figure 10a, where the eutectic silicon network consists of ultra-fine nano-eutectic silicon particles since the Al substrate is dissolved. After removing the corrosion products, the surface of the alloy is flat, where a convex network of small silicon particles (nano-size) becomes visible. This is because the potential difference between the nano-silicon particles and the substrate is low in the electrolyte, which causes the aluminum substrate to be slightly dissolved. Figure 10c,d show the morphology of Alloy E, where the second-phase silicon particles precipitated from the aluminum matrix are partly separated from the matrix and the corrosion behavior with significant pits and unevenness is observed. The micron-sized spherical Si particles (maximum about 3 µm) are isolated and uniformly distributed on the concave and uneven Al matrix. The aluminum matrix around the silicon particles is corroded to a large area because it serves as an anode. Figure 10e,f are the composition analysis of the silicon phase, which indicates that the left silicon phases after removing the corrosion products are, respectively, the eutectic silicon and the precipitated silicon particles.

**Figure 11:**
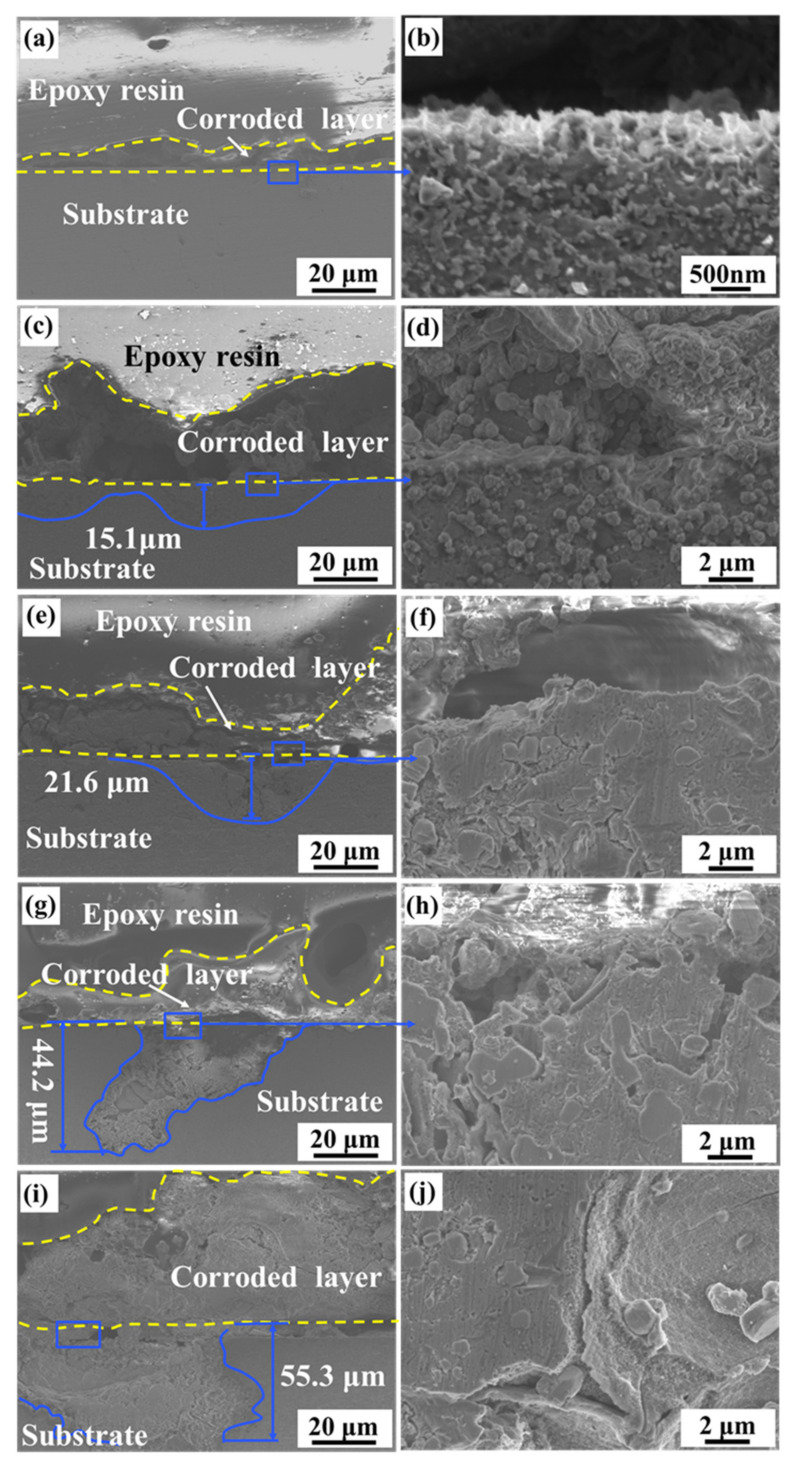
The morphology of the cross section of the alloys after corrosion: (**a**,**b**) Alloy A, (**c**,**d**) Alloy B, (**e**,**f**) Alloy C, (**g**,**h**) Alloy D, (**i**,**j**) Alloy E.

Figure 11 presents the cross-sectional morphology of Alloys A, B, C, D, and E after immersion for 40 days. Figure 11a provides a dense and complete corrosion product layer for Alloy A. Figure 11b is a partially enlarged morphology of the warped part of the corrosion product of Alloy A. The continuous eutectic silicon network on the surface facilitates the formation of a dense protective film, which prevents corrosion from spreading below the surface and delays the downward expansion process of corrosion. In other words, the continuous eutectic silicon network effectively slows down the corrosion process. Figure 11c,e,g,i are the corresponding cross-sectional morphologies of alloys B, C, D, and E, respectively. As the silicon particles grow, the eutectic silicon network is destroyed and granular silicon phases from small to large are precipitated. It leads to a corrosion product layer with uneven thickness, loose structure and distinctive cracks. As the silicon particles grow further gradually, the corrosion extension depth increases from 15.1 μm to 55.3 μm, and the corrosion breakdown tends more serious. Moreover, Figure 11f,h,j present that the Al matrix around the Si particles dissolves significantly and spreads downward severely as the precipitation of silicon particles grows. It is revealed that the growth of silicon particles can induce local intergranular corrosion to occur.

## 4. Discussion

### 4.1. Microstructure Transformation Morphology of Eutectic Silicon

The grain size distribution characteristics of alloys A and E are analyzed by EBSD. Their microstructures are composed of overlapping fan-shaped molten pools, where the interior of the molten pool is dominated by columnar grains, and the boundary of the molten pool is dominated by fine equiaxed grains. Firstly, the morphology of the grain structure is mainly affected by the temperature gradient (G) and the growth rate (R). The ratio of G/R controls the morphology of the microstructure at the solidification front. In the molten pool, G gradually decreases along the boundary of the molten pool toward the center of the molten pool, while R gradually increases. Therefore, the G/R ratio gradually decreases from the molten pool boundary to the molten pool interior, and grains morphology is transformed from columnar grains to equiaxed grains. In addition, a part of the area consists of a mixture of columnar and equiaxed grains, which mainly results from the existence of Marangoni convection in the liquid molten pool and the high thermal conductivity of the solidified AlSi10Mg alloy. Due to the complex changes of thermal boundary occurring near each adjacent molten pool, especially Marangoni convection, the G/R ratio is effectively changed, which influents the microstructure supercooling and stability of the solidification front. Hence, the morphology of solidified grains is changed from equiaxed grains to columnar grains. G × R represents the cooling rate and determines the size of the grain. There is no obvious difference in the overall microstructure of the materials, which indicates that the values of G/R and G × R are stable and consistent [33]. Secondly, the LAGB occupies the boundary position of the molten pool. It mainly results from the partial or full remelting of the previously solidified layer under the irradiation of the high-energy laser beam when preparing a new layer. However, many small equiaxed grains are observed in Alloy E, which results from the spheroidization of Si particles caused by the evolution between the Al/Si phases. Although the grain boundaries are observed on IPF, they are actually the precipitated fine spherical silicon particles, rather than the recrystallized equiaxed grains [17].

From further enlargement of the SEM morphology, the microstructure of different alloys shows a distinctive difference. For Alloy A, the size of the eutectic silicon network in different regions, such as the fine crystal region, coarse crystal region and heat-affected zone, was caused by the overlap during the preparation process. In the microstructure of the fine-grained region, the supersaturated eutectic Si is precipitated at the boundary of the cellular aluminum, which does not have time to grow up due to the high cooling rate of SLM technology of 10^6^~10^8^ K/s [34]. In coarse-grained regions, the overlapping leads to local heat treatment of the solidified melt channel and coarsening of the microstructure [35,36]. After heat treatment, the eutectic Silicon network is broken and evolves into spherical silicon particles. On one hand, the dislocation of atoms, crystal defects, and rounding of the particle tip during the heat treatment process generates homogenization energy [30]. On the other hand, the heat-driven irregular curvature improves the force field between the silicon particles and the matrix and reduces the surface energy by changing the shape of the silicon particles [37]. As the heat treatment time increases, the uniformly distributed Si particles decrease in number and increase in size. Upon high-temperature heat treatment, the large diffusion coefficient and large silicon concentration gradient of silicon promote the rapid diffusion of silicon atoms in the matrix. It makes silicon atoms diffuse and dissolve, resulting in a more uniform distribution in the matrix [38,39]. The decreasing number of silicon particles may be a result of particle coalescence and Ostwald ripening. Large particles grow at the expense of merging small particles [8,18]. Regarding the reason for the silicon particles size increasing, the external energy from heat treatment causes these solute atoms to be activated and segregated. It makes the solute atoms diffuse and migrate, and some of them migrate to low-energy positions. According to the principle of minimum interface energy, these fine Si phases are continuously moved toward a spherical evolution [40,41].

### 4.2. The Effect of Si Phase Morphology on Mechanical Properties

The microhardness of AlSi10Mg alloy significantly varies before and after heat treatment, where Alloy A has the highest microhardness. Due to the high cooling rate during the SLM preparation process. Silicon exists in a supersaturated state, where the honeycomb-like dendritic structure, dislocation structure and eutectic silicon network are embedded in the α-Al matrix as nanoparticles, playing the role of fine-grain strengthening. Moreover, there are solid solution strengthening and dispersion strengthening effects of Mg and Si elements, which are consistent with previous work [19]. As the time of heat treatment increases, the supersaturated Si atoms are precipitated from the Al matrix and the silicon particles coarsen, weakening the solid solution strengthening effect. Meanwhile, the distance between Si particles increases significantly, which makes its strength decrease. This result is also attributed to the coalescence of small silicon particles and Ostwald ripening, which leads to an increase in particle size and a decrease in number. Meanwhile, the increase in alloy ductility is attributed to the following reasons. Firstly, the decrease in the number of silicon particles and the increase in size lead to a decrease in local stress or strain. Secondly, the heat treatment reduces the residual stress during the SLM process [18].

### 4.3. The Effect of Si Phase Morphology on Corrosion Behavior

As discussed above, the size and morphology of the Si phase have a significant effect on the corrosion behavior of the AlSi10Mg alloy. For Alloy A, nano-eutectic silicon particles act as a micro-cathode on the surface of the alloy, which can promote the anodic dissolution of the matrix and the early formation of Al(OH)_3_. The network structure composed of nano-eutectic silicon particles facilitates the deposition of corrosion products and produces an effective barrier layer to prevent further corrosion of the matrix. Therefore, the AlSi10Mg alloy directly fabricated by SLM (Alloy A) has good corrosion resistance. However, the heat treatment causes the silicon particles to be gradually coarsened and to be homogeneously dispersed in the matrix. During the corrosion process, these homogeneously dispersed silicon particles act as a cathode, and the aluminum matrix acts as an anode, producing many galvanic couples on the surface of the alloy. The area ratio of the cathode and the anode in the aluminum alloy is the key factor to evaluate the corrosion rate [11,42]. Since the potential difference between the matrix and the grown silicon particles is higher than that of the nano-eutectic silicon particles, a micro-battery loop of “a large anode-a small cathode of silicon particles” is created between the matrix and the silicon particles. Meanwhile, the increased corrosion driving force causes the oxide film to fluctuate and form a loose oxide layer [43]. Therefore, the aluminum matrix around the silicon particles dissolves significantly by removing the corrosion products after heat treatment, resulting in severe surface damage. The second phase precipitated after the heat treatment is silicon particles, which causes the segregation and depletion of Si elements at the grain boundaries. The matrix in the Si-poor region and silicon particles constitute a concentration cell. Under the action of the corrosive electrolyte Cl^-^, the solute-poor region in the matrix is preferentially dissolved [44]. It causes the corrosion to extend along the solute-poor region around the Si particles, and intergranular corrosion occurs in alloys.

**Figure 12:**
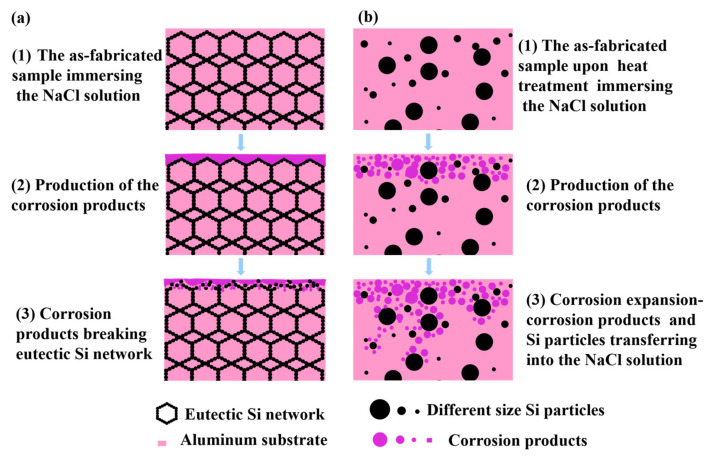
Schematic illustration of corrosion process of AlSi10Mg alloy fabricated by SLM and the heat-treated alloys in the 3.5 wt.% NaCl solution: (**a**) AlSi10Mg alloy, (**b**) the heat-treated alloy.

Figure 12 illustrates the corrosion process of different silicon phase sizes and morphologies. For Alloy A (the AlSi10Mg alloy directly fabricated by SLM), the potential difference generated between the ultra-fine nano-eutectic silicon particles and the Al matrix is small. Meanwhile, the corrosion products are deposited and grow in the eutectic silicon network to produce a dense protective film, which acts as a barrier to corrosive ions and hinders the expansion of corrosion, as shown in Figure 12a. After the heat treatment, a large potential difference is generated between the coarsen Si particles and the matrix, which makes the chemical reaction more active. The corresponding microstructure is not conducive to the deposition of corrosion products, resulting in a loose and porous oxide layer. Meanwhile, the corrosion products and silicon particles fall off and accelerate the expansion of corrosion, then the corrosive ions cause intergranular corrosion along the grain boundaries, as shown in Figure 12b. As the corrosion product grows up and destroys the eutectic silicon network, the corrosion in Alloy A spreads slower compared with alloys after heat treatment. Hence, the AlSi10Mg alloy directly fabricated by SLM (Alloy A) has better corrosion resistance.

## 5. Conclusions

The microstructure, mechanical properties and corrosion properties of AlSi10Mg alloy fabricated by SLM before and after heat treatment are investigated. The main conclusions are as follows:

(1) The difference in the average grain size and the proportion of large-angle grain boundaries between Alloy A (the SLM directly fabricated AlSi10Mg alloy) and Alloy E (the alloy after heat treatment at 500 °C/5 h) are not distinctive, which indicates that the heat treatment cannot change the grain size and grain boundary distribution of the AlSi10Mg alloys. After heat treatment, the microstructure of the AlSi10Mg alloy is transformed from a continuous network structure with nano-eutectic silicon particles to coarse and homogeneously distributed isolated silicon particles. Moreover, the heat treatment reduces the hardness of AlSi10Mg alloys.
(2) With the coarsening of silicon particles, the corrosion current density increases from 3.39 × 10^−7^ A·cm^−2^ to 5.01 × 10^−6^ A·cm^−2^, which means the corrosion resistance gradually deteriorates. Based on qualitative and quantitative analysis, the heat treatment reduces the corrosion resistance of SLM-fabricated AlSi10Mg alloys. The corrosion mechanism changes from local pitting to a combination of pitting and intergranular corrosion. Moreover, the directly fabricated alloy corrodes preferentially in the overlapping region of the boundary of the molten pool.
(3) The silicon phase has a dual effect on the corrosion behavior of AlSi10Mg alloys. On one hand, it affects the corrosion driving force on the surface. The electrical potential difference generated by the nano-eutectic silicon particles on the surface is small, which leads to a low chemical reaction activity and an easy formation of a dense protective film. On the other hand, the networked distribution of eutectic silicon benefits the deposition of corrosion products, thereby playing a protective role and slowing down alloy corrosion. With the silicon particles coarsening, the corrosion driving force between the silicon particles and the matrix increases, generating a loose and porous oxide film on the surface. Meanwhile, the corrosion products on the surface of the isolated silicon particles are easily transferred to the solution, which is not conducive to the deposition of corrosion products and weakens the alloy’s corrosion resistance.

## Figures and Tables

**Table 1:** Chemical composite of the AlSi10Mg Powder (wt.%).

Si	Mg	Cu	Ni	Fe	Mn	Ti	Al
10	0.4	<0.25	<0.05	<0.25	<0.1	<0.15	Bal.

**Table 2:** Parameters of SLM process.

Process Parameter	Laser Power	Exposure Time	Spot Size	Layer Thickness	Point Distance	Atmosphere
Value	300 W	140 μs	70 μm	30 μm	0.3	Argon

**Table 3:** The holding heat treatment times of AlSi10Mg fabricated by SLM.

Alloys	Heat Treatment Process
Alloy A	As-fabricated SLM
Alloy B	500 °C × 15 min
Alloy C	500 °C × 1 h
Alloy D	500 °C × 2 h
Alloy E	500 °C × 5 h

**Table 4:** The EDS point scan analysis results of the typical positions in Figure 4 (at. %).

Point No.	1	2	3	4	5
Al	84.11	16.98	13.10	13.00	10.20
Si	15.89	83.02	86.90	87.00	89.80

**Table 5:** The electrochemical fitting parameters of alloys A–E.

Sample	*Icorr* (A·cm^−2^)	*Ecorr* (V)
Alloy A	3.39 × 10^−7^	−0.81
Alloy B	6.92 × 10^−7^	−0.88
Alloy C	1.58 × 10^−6^	−0.90
Alloy D	2.51 × 10^−6^	−0.83
Alloy E	5.01 × 10^−6^	−0.91

**Table 6:** The fitting results of EIS of Alloys A-E in the aerated 3.5 wt.% NaCl solution.

Samples	R_f_ (kΩ·cm^2^)	CPE_f_ × 10^−5^ (S·s^n^)	*n*	R_t_ (kΩ·cm^2^)	CPE_t_ × 10^−4^ (S·s^n^)	*n*	W × 10^−2^ (S·s^0.5^)	Chi-Square
Alloy A	15.70	1.41	0.82	45.12	8.14	0.80	4.35	1.70 × 10^−3^
Alloy B	7.62	2.43	0.67	9.11	3.03	1	17.59	6.32 × 10^−3^
Alloy C	1.98	1.51	0.82	3.05	3.06	0.47	5.07	4.46 × 10^−3^
Alloy D	1.69	6.89	0.64	2.35	1.73	0.78	30.19	2.50 × 10^−3^
Alloy E	1.11	6.69	0.67	2.07	1.89	0.77	0.45	2.49 × 10^−3^

**Table 7:** EDS point scan analysis results of the typical positions in Figure 9 (at.%).

Atoms	Position A	Position B	Position C	Position D	Position E
Al	57.4	76.1	62.5	55.0	71.4
O	33.6	13.2	26.4	34.1	19.0
Si	8.7	8.6	8.8	7.7	8.8
Cl	0.4	2.1	2.2	3.2	0.9

## Data Availability

All data generated or analyzed during this study can be made available by the corresponding author upon request.
